# Recent Developments in Paper-Based Sensors with Instrument-Free Signal Readout Technologies (2020–2023)

**DOI:** 10.3390/bios14010036

**Published:** 2024-01-11

**Authors:** Danni Yang, Chengju Hu, Hao Zhang, Shan Geng

**Affiliations:** 1 Chongqing Engineering Research Center of Pharmaceutical Sciences, Chongqing Medical and Pharmaceutical College, Chongqing 401331, China; 2220089@cqmpc.edu.cn; 2 Health Management Center, The Affiliated Dazu Hospital of Chongqing Medical University, Chongqing 402360, China; 13752933067@163.com; 3 Department of Endocrinology, The Affiliated Dazu Hospital of Chongqing Medical University, Chongqing 402360, China

**Keywords:** paper-based sensors, instrument-free signal readout, distance-based, counting-based, text-based

## Abstract

Signal readout technologies that do not require any instrument are essential for improving the convenience and availability of paper-based sensors. Thanks to the remarkable progress in material science and nanotechnology, paper-based sensors with instrument-free signal readout have been developed for multiple purposes, such as biomedical detection, environmental pollutant tracking, and food analysis. In this review, the developments in instrument-free signal readout technologies for paper-based sensors from 2020 to 2023 are summarized. The instrument-free signal readout technologies, such as distance-based signal readout technology, counting-based signal readout technology, text-based signal readout technology, as well as other transduction technologies, are briefly introduced, respectively. On the other hand, the applications of paper-based sensors with instrument-free signal readout technologies are summarized, including biomedical analysis, environmental analysis, food analysis, and other applications. Finally, the potential and difficulties associated with the advancement of paper-based sensors without instruments are discussed.

## 1. Introduction

Paper-based sensors have become increasingly popular for a range of analytical applications in recent years due to their convenience, affordability, portability, and user-friendliness. Since the paper-based sensors were first reported in 2007 [1], they have been widely employed in biomedical analysis [2,3], environmental pollutant tracking [4,5], food analysis [6], and other fields [7] for many years. Nevertheless, these quantitative processes typically involve a range of technologies such as electrochemistry [8], fluorescence [9], chemiluminescence [10], or electrochemiluminescence [11], which usually require some specialized instrument and skilled personnel. Thus, developing quantitative analysis techniques that do not require specific instruments yet still provide precise and dependable results is a challenge in the application of paper-based sensors.

Instrument-free readout technologies are indispensable for increasing the usability and accessibility of paper-based sensors. Traditional spectroscopic analysis technologies rely on the variations in color difference or intensity before and after the reaction to perform a qualitative or quantitative analysis of the analyte. This process, however, necessitates the use of specialized instrumentation [12], which can be inconvenient and inefficient. Paper-based colorimetric assays rely on the color change caused by the interaction between the chromogenic reagent and the analyte for instrument-free visual qualification or quantification. A variety of paper-based sensors with instrument-free signal readout have been developed, including the transformation of the detection signal into a measurement of color distance changes [13], counting the number of color change areas [14], readout of display text [15], or other technologies [16], which can be directly or indirectly quantified or semi-quantitatively assessed with the naked eye.

**Figure 1:**
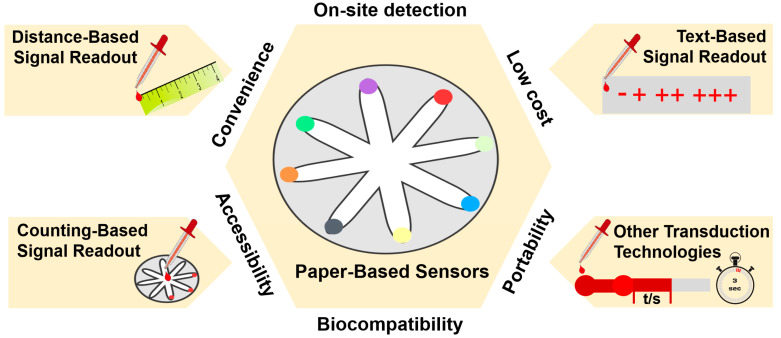
Schematic of paper-based sensors with instrument-free signal readout technologies.

Paper-based sensors with instrument-free signal readout have been gaining attention as a technology that could be utilized for a variety of point-of-care testing applications. To date, there have been several reviews on paper-based sensors, mainly focusing on distance-based microfluidics [17,18]. Moreover, the applications of paper-based sensors with instrument-free signal readout have been reviewed in 2019 by Li et al. [19]. In this review, we focus on the different instrument-free signal readout technologies, including distance-based signal readout technology, counting-based signal readout technology, text-based signal readout technology, and other transduction technologies in the last three years (2020–2023) (Figure 1). Then, the applications of paper-based sensors with instrument-free signal readout are summarized, including biomedical analysis, environmental analysis, food analysis, and other applications. Finally, the potential and difficulties associated with the advancement in paper-based sensors without instruments are discussed.

## 2. Instrument-Free Signal Readout Technologies of Paper-Based Sensors

Recent progress in instrument-free signal readout technologies has enabled the simplification of the use and acquisition of paper-based sensors. The technologies for signal acquisition can be classified into four general categories: the distance-based signal readout technology utilizes migration distance of color change; the counting-based signal readout technology relies on numerical counting or quantification of visual indicators; the text-based signal readout technology utilizes printed texts; and other transduction technologies explore alternative detection technologies. These technologies, which are simple and cost-effective, offer great potential for applications in various fields that require rapid and on-site analysis.

### 2.1. Distance-Based Signal Readout Technology

This technique achieves instrument-free signal readout on a paper-based sensor by quantifying the change in solution migration distance caused by the analyte. The paper substrate is often equipped with hydrophilic channels or patterned areas to facilitate the rapid flow of liquids, thereby increasing the efficiency of sample transfer. Additionally, the use of patterned areas on the paper substrate allows for a more uniform distribution of the sample, resulting in more accurate results. As the sample solution containing the analyte walks through these channels or areas via capillary action, a visible color change occurs due to chemical reactions between the analyte and reagents impregnated in these regions. Finally, quantitative analysis can be accomplished without any apparatus by assessing the migration distance of color change in combination with a previously established calibration curve.

**Figure 2:**
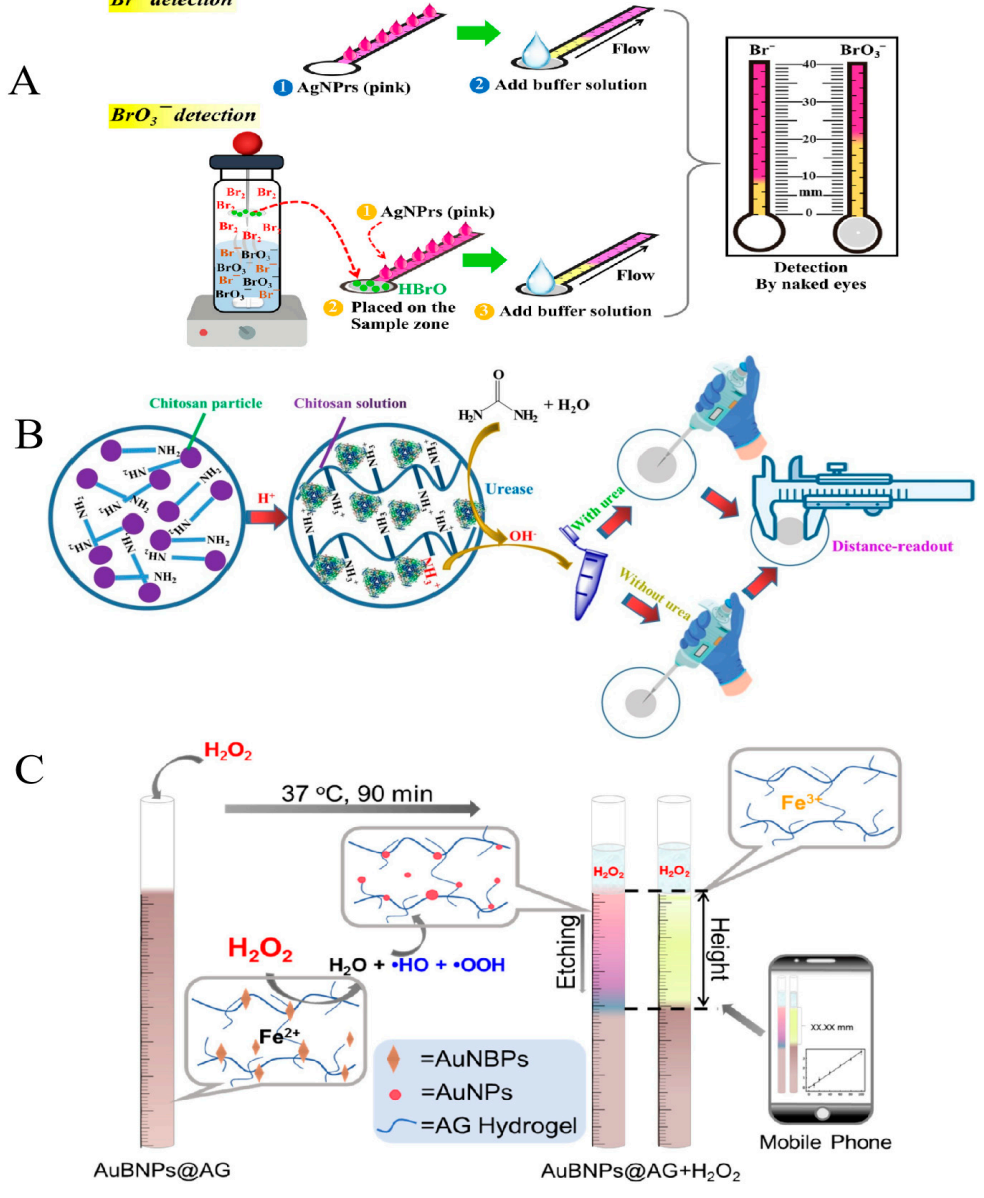
(**A**) Schematic of length distance-based instrument-free signal readout technology for the detection of Br^−^ and BrO_3_^−^. Reprinted with permission from Ref. [22]. (**B**) Schematic of radial distance-based instrument-free signal readout technology. Reprinted with permission from Ref. [23]. (**C**) Schematic of height distance-based instrument-free signal readout technology. Reprinted with permission from Ref. [25].

To obtain a more convenient and less resource-consuming quantitative method, a distance-based signal readout technology, first reported in 2013 [20], is used to measure glucose, nickel, and glutathione, respectively. Since then, numerous distance-based instrument-free signal readout techniques that make use of length measurements have been widely established. For instance, Ogawa et al. [21] designed a foldable origami paper-based device that utilizes the molybdenum blue reaction to enable the semi-quantitative monitoring of ionic silica. Initially, the modified paper strips were treated with cationic poly(allylamine hydrochloride) followed by indicator heptamolybdate, and then the analytes containing ionic silica were pipetted into the reduction area for 30 min, a yellow silicomolybdic complex was reduced by ascorbic acid and turned blue. The ionic silica sample was detectable between 50 mg/L and 1000 mg/L with the lowest detection level being 50 mg/L. In addition, Phoonsawat et al. [22] developed silver hexagonal nanoprism (AgNPrs)-modified distance-based paper sensors with a view to achieving high levels of sensitivity and selectivity for identifying the presence of Br^−^ and BrO_3_^−^ (Figure 2A). The concentration of BrO_3_^−^ can be determined by coupling a paper-based sensor with a headspace extractor method to convert it to Br_2_. The detection of Br^−^ involves its conversion to Br_2_ in the presence of oxygen, which then quickly transforms into the strong oxidizing agent HBrO of AgNPrs when mixed with water. The AgNPrs (pink color) react with the BrO_3_^−^ obtained via headspace extraction and the Br^−^ in solution to produce silver nanospheres (yellow color) and AgBr through an oxidative process. Finally, the yellow bands can be used to quantify Br^−^ and BrO_3_^−^ by visually measuring their lengths. This method enables the analysis of Br^−^ and BrO_3_^−^ at concentrations ranging from 25 to 2000 μg/L and 0.5 to 50 μg/L, respectively. The limit of detection (LOD) for Br^−^ was 10 μg/L, while that for BrO_3_^−^ was 0.5 μg/L.

Another distance-based instrument-free signal readout technology by measuring the radial distance has also attracted attention. That is, the sample solution undergoes radial diffusion on the filter paper via capillary force, leading to the formation of a circular colored spot. The radius of the circular spot is in direct correlation to the quantity of the analyte (Figure 2B). A radial distance-based instrument-free signal readout technology was developed by Zhang et al. [23] to measure urea concentration based on the pH-dependent and high viscosity variation characteristics of chitosan in a solution state. The measurement of urea levels in this study can be accomplished by utilizing urease, an enzyme that catalyzes the decomposition of urea into ammonia, thereby elevating the pH of the solution and altering the viscosity of chitosan. Finally, the amount of urea is connected to the radial diffusion distance on the paper substrate, which can be ascertained with a vernier caliper. Under optimal conditions, the developed method is capable of analyzing urea concentrations between 3.8 mM and 15.1 mM, with a limit of quantitation (LOQ) of 3.8 mM. In addition, a similar study was also reported by Zhang et al. [24] to measure glucose concentration using a glucose oxidase-catalyzed reaction to cause a decrease in the solution’s pH to release calcium ions from calcium carbonate, and the released calcium ions combined with sodium alginate to form a calcium alginate hydrogel, thereby increasing the viscosity of the solution. Finally, a vernier caliper was employed to measure the radial diffusion diameter of the mixed solution that was pipetted onto the filter paper. Employing this approach, glucose concentrations between 1.4 mM and 7.0 mM can be analyzed, with an LOQ of 1.4 mM.

Apart from the above technologies, a distance-based instrument-free readout technology for measuring height has been reported by Shou et al. [25], who created a multicolored hydrogel sensor with the aim of recognizing H_2_O_2_ through the process of etching gold nanobipyramids (AuNBPs) (Figure 2C). The introduction of H_2_O_2_ caused a marked alteration in the color of AuNBPs@agarose hydrogel as the concentration of H_2_O_2_ increased. Finally, the height of the color change reflected the amount of H_2_O_2_ in the solution. It was observed that a linear relationship existed within ranges of 20–100 μM, 200–1000 μM, 1–10 mM, and 10–100 mM when this approach was utilized. Signal readout technology based on length measurement, which does not require modification of the paper substrate, is suitable for detecting analytes that cause changes in solution viscosity. On the other hand, signal readout technologies based on radial distance and height require modification of the paper substrate and are suitable for detecting analytes that trigger color development reactions.

### 2.2. Counting-Based Signal Readout Technology

Digital counting or quantification of visual indicators is used in counting-based signal readout technology to obtain signal readout from paper-based sensors. This approach usually involves incorporating indicator molecules that undergo specific reactions with the target analytes, resulting in color changes or the formation/disappearance of distinct patterns or symbols. The amount of occurrences or presence/absence of these indicators is then related to the quantity of the analyte through the quantitative relationships or the established standards.

**Figure 3:**
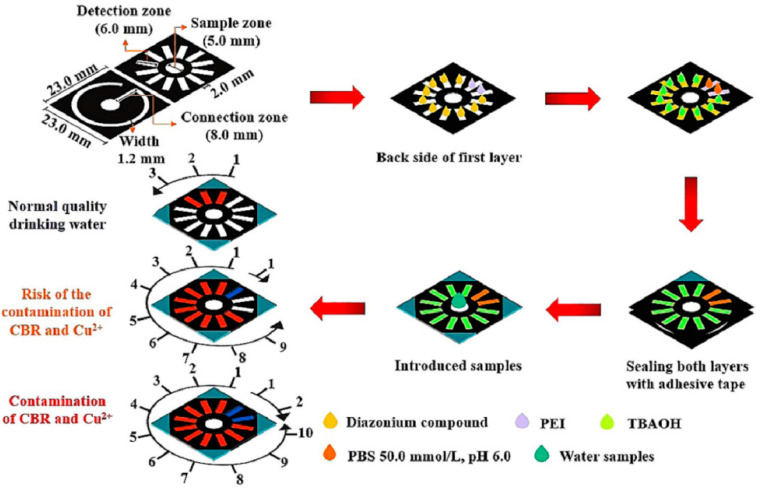
Schematic of counting-based instrument-free signal readout technology. Reprinted with permission from Ref. [27].

The counting-based signal readout sensor was first reported by Lewis et al. [26]. In this study, the number of colored bars can be employed to identify the amount of H_2_O_2_. A similar study was also reported by Khachornsakkul et al. [27], who employed a novel counting-based instrument-free signal readout device designed to detect the Cu^2+^ and carbaryl (CBR) in real samples. The paper analytical device, depicted in Figure 3, is constructed with two layers of filter paper; the initial layer is equipped with twelve detection zones, all of which have been pre-loaded with specific reagents to measure Cu^2+^ and CBR, and the subsequent layer is used to establish a pathway between the sample and detection zones. After the introduction of samples containing Cu^2+^ or CBR, they can react with the reagent that had been previously deposited and produce a color change in each successive zone. The concentration of Cu^2+^ and CBR can be ascertained by measuring the number of blue and orange detection areas, respectively. Finally, this method has demonstrated its capacity to analyze Cu^2+^ and CBR in the ranges of 1.25–15.0 μg/mL and 0.01–0.12 μg/mL, with an LOQ of 1.25 μg/mL and 0.01 μg/mL, respectively. To overcome the challenges of using sub-microliter sample volumes for semi-quantitative assay, a paper-based microfluidic sensor was innovatively created to enable the semi-quantitative detection of both immunoglobulin (IgE) and glucose through the use of a counting-based lateral flow assay [28]. In this study, three points along the sample flow path were supplied with 0.2 μL of the assay reagent, resulting in a count-based colorimetric assay signal. After the introduction of the analyte, they can react with the reagent that had been previously deposited at the test points and produce a color change at each point. The levels of IgE and glucose can be determined by counting the colored points. Through the developed method, it is possible to analyze IgE and glucose concentrations ranging from 0 to 400 ng/mL and 0 to 12 mmol/L, respectively.

### 2.3. Text-Based Signal Readout Technology

Text-based signal readout techniques necessitate the use of paper-based sensors that have been printed or pre-loaded with text. This process typically requires the addition of a reagent that interacts with the substance being analyzed, leading to the production of a distinct textual response, such as a number, letter, or symbol. Through visual comparison of the generated text with a reference scale, or by interpreting its variations, quantitative data can be obtained without the requirement of any additional apparatus.

**Figure 4:**
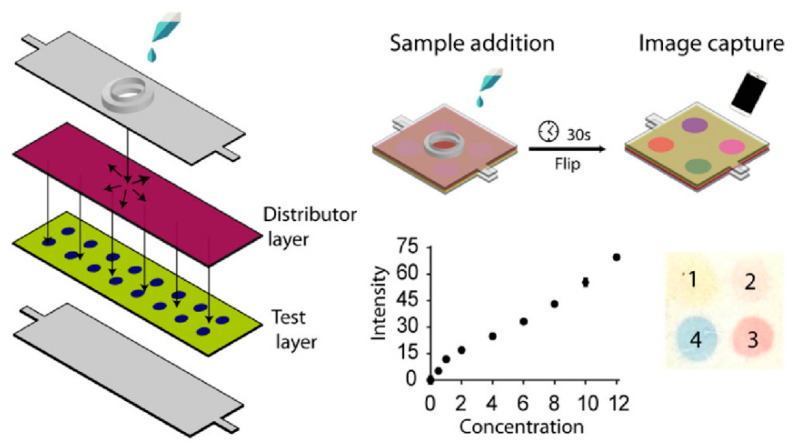
Schematic of text-based instrument-free signal readout technology. Reprinted with permission from Ref. [30].

The text-based signal readout technology necessitates the use of pre-patterned on a paper substrate, such as those with and without obstacle patterns, which are usually produced through inkjet printing and pen writing. For example, Yamada et al. [29] designed a paper-based text-displaying sensor involving an obstacle pattern for the semi-quantitative detection of human serum albumin. The developed method allows for a direct readout of the values without the need for matching the colors to a reference chart, thus decreasing the chance of an inaccurate assessment. Despite the simplicity and convenience of text-based signal readout technology being based on an obstacle-involved pattern, the need for pre-loaded obstacles to form the flow channels renders the mass production of the patterned paper a laborious process. To overcome this problem, Chauhan et al. [30] designed a paper-based microfluidic sensor without any obstacle pattern that can recognize salivary thiocyanate, proteins, glucose, and nitrite via colorimetric analysis (Figure 4). The sensor is constructed from two layers: a liquid distribution layer and a reagent layer for detection, both of which have different wicking rates. In this study, a pH indicator containing bromophenol blue solution was first deposited on the detection layer, and then the distribution, detection, and base layers were bonded together. Subsequently, the clinically relevant analytes were introduced into the sample wells and diffused from the diffusion layer to the detection layer using different adsorption rates. Finally, an image analysis software was used for quantitative and semi-quantitative analysis.

### 2.4. Other Transduction Technologies

Besides the technologies mentioned above, other transduction techniques have been documented for instrument-free signal readout in paper-based sensors such as time-based and self-powered signal readout technologies. Wu et al. [16] developed a quantitative thrombin assay using a time signal output technique. In this study, the thrombin aptamer was demonstrated to be an effective method for the accurate detection of thrombin, due to its capability to transition from a loose form to a rigid G-quadruplex structure, thus partially concealing the surface of the Au NPs. Thrombin could be determined between 1.3 nM and 43 nM with an LOD of 0.9 nM. In addition, Liu et al. [31] designed an origami paper-based device, powered by itself, which can detect adenosine-5′-triphosphate (ATP) through a reaction initiated by glucose oxidase (GOx). Firstly, a sandwich structure is formed in the detection zone via the combination of DNA sequence, aptamer, and GOx modified by DNA2. Subsequently, the binding between ATP and aptamers is activated when varying concentrations of ATP are added, which in turn causes the liberation of GOx-DNA. This released GOx-DNA then traverses the hollow region to the reaction zone, where it catalyzes glucose oxidation and produces electrons, thus changing the amount of K_3_[Fe(CN)_6_]. The proposed device has the capability to identify ATP concentration in the interval of 10–5000 nM.

## 3. Application of Paper-Based Sensors with an Instrument-Free Signal Readout

Over the last few years, paper-based sensors have gained increasing attention, with a range of paper-based analytical tools being designed for different applications. The reasons for this are as follows: (1) paper is one of the cheapest and most unique materials; (2) based on different design principles such as surface wettability, viscosity, flow control, and colorimetry [32,33,34,35], as well as preparation technologies, including screen printing, laser printing, and wax printing [36,37,38], paper or modified paper have been used as microreactors with specific functions and to realize the detection of different analytes. Currently, paper-based sensors with instrument-free signal readout technologies are a popular choice in biomedical analysis, environmental pollutant tracking, food analysis, and other aspects.

### 3.1. Biomedical Analysis

Paper-based sensors, which do not require instrument signal readout technologies, are popular for their cost-effectiveness, ease of use, and portability in biomedical analysis, especially in monitoring physiological parameters and detecting biomarkers. The application of paper-based sensors with instrument-free signal readout technologies in biomedical analysis is shown in Table 1.

Cancer is a crucial global health issue that is currently affecting people everywhere. Tumors are formed when cancer cells grow abnormally and uncontrollably. Traditionally, cancer diagnosis was carried out using nuclear magnetic resonance, or computed tomography, which relies on some special instruments and professional operators. Therefore, recognizing cancer in its early stages is vital for decreasing the severity of the illness and improving the quality of life for patients. The detection of relevant biomarkers during cancer development can be used in the diagnosis and treatment of cancer. MicroRNA, which is released from cells and organs, has been determined to be strongly associated with the occurrence and growth of cancer and is thus being used as a cancer biomarker. Zhang et al. [51] combined catalytic hairpin assembly (CHA) and hybridization chain reaction (HCR) techniques to develop a paper substance sensor for the quantitative measurement of miRNA-21 based on distance signal readout. In the presence of miRNA, CHA initiates HCR amplification to generate substantial amounts of G-quadruplex, which binds to heme to produce the Hemin/G-quadruplex peroxidase-mimicking DNAzyme. This peroxidase-mimicking then catalyzes the transformation of iodide to iodine when it is mixed with H_2_O_2_. As a result of the starch–iodide reaction, the paper shows a bright blue/purple-hued band that can be accurately determined using a ruler. Finally, the proposed method is capable of detecting miRNA-21 concentrations between 1.0 pM and 1000 pM, with an LOD of 0.2 pM. Tai et al. [52] created a paper-based sensor that interprets the changes in viscosity brought about by the breakdown of hydrogel into a visual signal that is determined by the distance. This study has shown that combining Cu^2+^ and alginate can create a copper-alginate hydrogel, and the presence of pyrophosphate ions has a strong attraction to Cu^2+^ that causes the gel–sol transition. The hydrogel’s disintegration alters the viscosity of the solution, which is then reflected in a shift in the distance on pH test papers, thus allowing for the identification of ALP and alpha-fetoprotein. Finally, the proposed method enables the measurement of ALP from 1 to 100 mU/mL with an LOD of 1.7 mU/mL, and alpha-fetoprotein from 0.5 to 30 ng/mL with an LOD of 0.8 ng/mL.

Viruses, as a widespread biological entity in nature, especially in the past three years, have posed a major security threat to the world with the emergence of COVID-19. Qian et al. [40] proposed a combination of the manual centrifugal micropipette tip approach and distance-based signal readout to facilitate the identification of SARS-CoV-2. When viral antigens were present, nanoparticles encapsulating monoclonal antibodies coalesced to form larger complexes. After incubation and centrifugation, the complexes that have formed aggregates settle out of the solution, while the free nanoparticles remain in suspension due to their lower density. Finally, quantitatively measuring the length of the aggregates that have been formed is easily achieved with visual inspection. The proposed strategy has the capacity to recognize SARS-CoV-2 viral antigens at a level of 1 ng/mL, with a linear range between 1 ug/mL and 10 μg/mL.

Diabetes, a metabolic disease that is currently widely prevalent in the world, is mainly caused by abnormal glucose metabolism of the organism itself and is also clinically diagnosed mainly by detecting the glucose content in the blood. Paper-based sensors with a distance-based signal readout have also been widely used for glucose determination. Allameh et al. [41] employed a cellulose paper-based sensor constructed by a carbon dioxide laser to visually detect the concentration of glucose in tear fluid. To complete the process, the first circular area should be filled with a solution containing GOx and HRP, while the detection channel should be supplemented with a solution of 3,3′,5,5′-tetramethylbenzidine (TMB) that is able to color the development. When a sample containing glucose is added to the sample area, it migrates to the first circular area via capillary action and interacts with GOx to produce hydrogen peroxide, which then reacts with the TMB on the detection channel with the help of HRP to produce a hued band related to the glucose level. Results indicated a linear response from the distance-based paper sensors when testing glucose levels from 0.1 mM to 1.2 mM, with an LOD of 0.1 mM. In addition, a paper-based device was designed by Prapaporn et al. [53], which incorporated a distance signal readout and nanocellulose filter to identify glucose levels. Through catalysis, GOx facilitates the transformation of glucose to H_2_O_2_, which then causes the silver nanoparticle to lose its purple hue and become colorless. Glucose was detectable in the interval between 2.5 mM and 30 mM, with the lowest amount that could be detected being 0.1 mM.

**Figure 5:**
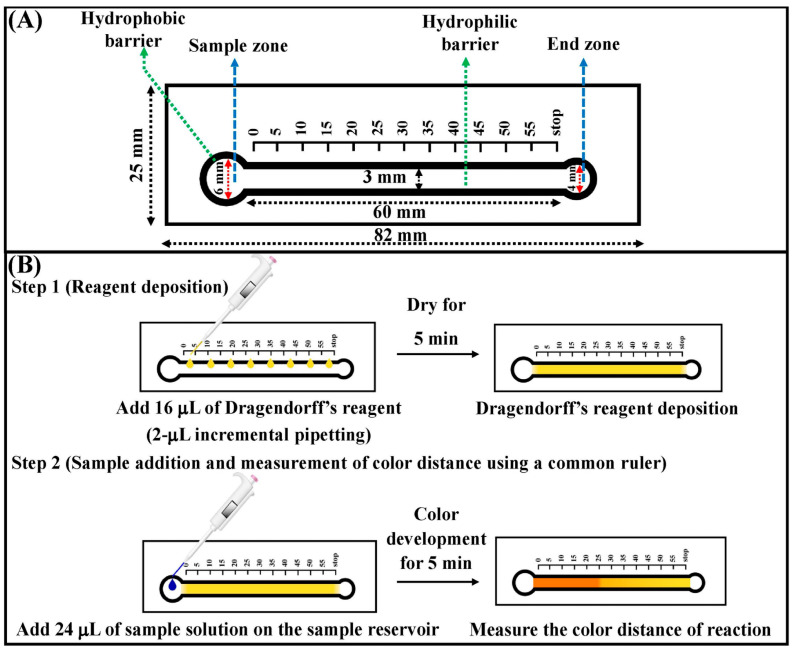
(**A**) The size of the distance-based paper sensor. (**B**) Schematic of distance-based instrument-free signal readout technology for the detection of sibutramine. Reprinted with permission from Ref. [54].

Moreover, Karamahito et al. [54] designed a filter paper device that utilized the principle of distance signal readout to quantitatively measure the amount of sibutramine present in slimming products, which could be also identified through visual assessment (Figure 5). The presence of sibutramine in a sample can be determined by the formation of an orange-red precipitate when reacted with Dragendorff’s reagent. The length of the orange-red complex was gauged with a ruler and was found to be proportional to the amount of sibutramine. Sibutramine was detectable in the interval between 0.22 mM and 0.9 mM, with the lowest amount that could be detected being 0.22 mM. Ping et al. [55] prepared a portable paper-based hydrogel sensor to recognize trypsin and its inhibitor. In this study, the gelatin hydrogel is capable of being hydrolyzed by trypsin, leading to the liberation of the water molecules that were entrapped within the hydrogel. The incorporation of a hydrogel mixture onto the surface of the pH test papers enabled the quantification of trypsin and its inhibitor aprotinin by utilizing a smartphone to measure the lateral flow distance. Finally, it is possible to detect trypsin quantitatively in the ranges from 1 × 10^−6^ to 1 × 10^−3^ mg/mL, with an LOD of 1.0 × 10^−6^ mg/mL. The measurement of the IC_50_ of aprotinin yielded a value of 11.07 ± 0.64 μg/mL.

### 3.2. Environmental Analysis

Environmental pollution has become an important threat to the construction of a green Earth. With the intensification of human activities and the development of industry, more and more pollutants are being directly released into the environment, especially the pollution of metal ions in water. Therefore, the rapid and accurate analysis of these environmental pollutants has become an increasingly important focus for scientists. The application of paper-based sensors with instrument-free signal readout technologies in environmental analysis and food safety is shown in Table 2.

**Figure 6:**
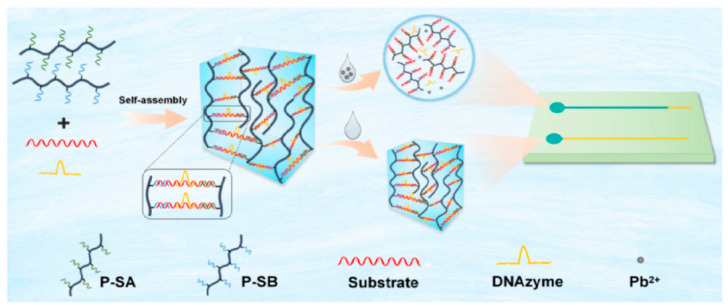
Schematic of distance-based instrument-free signal readout technology for the determination of Pb^2+^. Reprinted with permission from Ref. [66].

Liu et al. [66] designed a paper-based sensor that utilizes a distance-based signal readout to detect Pb^2+^ (Figure 6). The incorporation of Pb^2+^ into the DNAzyme caused the DNA hydrogel to disintegrate, thus allowing for water molecules to be transported on the patterned paper via capillary forces. The water flow distance was correlated with the amount of Pb^2+^ concentration. The lowest concentration of Pb^2+^ that could be identified is 3.0 nM. Ninwong et al. [67] established the potential of using a fluorescent paper-based device along with an evaporative pre-concentration system for the identification of Hg^2+^. In this study, nitrogen-doped carbon nanotubes (NCDs) were employed to interact with Hg^2+^ to form Hg^2+^-NCDs complexes, which in turn caused the fluorescence of the NCDs to be quenched, and the level of Hg^2+^ can be quantified by measuring the distance signal of the quenched fluorescence. The LOD of Hg^2+^ was 5 μg/L by the naked eye. To facilitate the analysis of K^+^ and Cl^−^, Phoonsawat et al. [68] developed a potentiometric ion-selective electrode that incorporated distance-based colorimetric detection. In this study, the concentration range of K^+^ and Cl^−^ ions that could be accurately measured were 0.1–100 mM and 0.5–50 mM, respectively, with LODs of 0.01 mM and 0.16 ± 0.05 mM. Nguyen et al. [60] also designed two kinds of paper-based devices coupled with the distance-based signal readout (“chemometer” format and radial design) for the quantitative of Al^3+^. The chemometer has the capacity to identify a concentration of 2.5 ppm (100 μM) and its linear range is between 2 and 54 ppm (100 μM–1 mM). Similarly, the radial device is able to recognize a concentration of 0.9 ppm (33 μM), and its linear range is between 2 and 24 ppm (100–900 μM).

### 3.3. Food Analysis

Food safety is a fundamental requirement for human sustenance, particularly in developing countries. Therefore, the development of a portable, economical, and user-friendly food safety testing platform is imperative. The application of paper-based sensors with instrument-free signal readout in food analysis is shown in Table 2.

**Figure 7:**
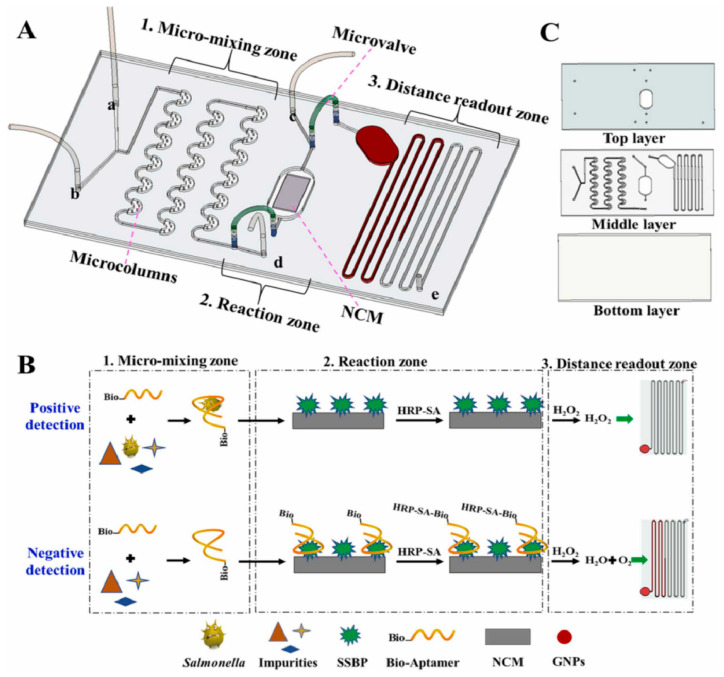
Schematic illustration of the principle of distance-based microfluidic aptasensor for the visual quantitative detection of *Salmonella*. (**A**) Structure and functional area distribution of a distance-based microfluidic aptasensor. (**B**) The detection principle of a distance-based microfluidic aptasensor. (**C**) The structure of the designed microfluidic chip. Reprinted with permission from Ref. [70].

A paper-based lateral flow sensor utilizing the variation in viscosity of pectin was developed by us [61] to identify the activity of polygalacturonase in cucumber. In this study, the hydrolysis of pectin by polygalacturonase alters its viscosity, which is evaluated with the aid of a smartphone by means of the diffusion distance on the pH test papers. Finally, polygalacturonase can be quantified between 0.025 and 0.80 U/mL, with an LOQ of 0.025 U/mL. To improve the reproducibility of the deposited sample volume, Al-Jaf et al. [62] developed a paper-based sensor that utilizes a ratiometric distance-based signal readout method to detect ascorbic acid with good precision and accuracy. To enhance the detection resolution and avoid excessive mixing of reagents, a three-dimensional spacing was established between the sampling and detection zones. The mixture solution containing Fe^3+^ and 1,10-phenanthroline, and the oxidized 3,3′,5,5′-tetramethylbenzidine were deposited in the first and second channels, respectively. Upon the addition of ascorbic acid, the color of the first channel shifted from a pale yellow to an orange-red, while the second channel changed from a greenish blue to colorless. Finally, the concentration of ascorbic acid can be measured between 0.05 mM and 1.2 mM, with an LOQ of 16 μM. In addition, Katelakha et al. [69] proposed a paper-based analytical tool that utilizes a distance-based signal readout to recognize lead ions in food matrices. This tool functions by taking advantage of the competitive binding between lead ions and carminic acid (CA) and polyethyleneimine (PEI). When a combination of lead ions and CA is introduced into PEI-modified paper, the length of red deposits along the flow path is reduced, as the lead ions are bound by both PEI and CA competitively. Finally, the detection of lead ions was found to be linear in the range of 5 µg/mL to 100 µg/mL, with the lowest detectable amount being 12.3 µg/mL. *Salmonella* is a common contaminant in various food items, including meat, eggs, milk, and vegetables. Man et al. [70] introduced a distance-based microfluidic aptasensor that is portable and can be used to quantitatively detect *Salmonella* (Figure 7). In this study, biotin-modified aptamers (bio-aptamers) were employed as recognition elements for *Salmonella typhimurium* to prepare bio-aptamer single strand-binding protein-modified nitrocellulose membranes. The binding of horseradish peroxidase-conjugated streptavidin to the modified nitrocellulose membrane initiates the decomposition of H_2_O_2_, thereby releasing O_2_ and propelling the gold nanoparticles forward. Thus, the distance signal readout can be used to determine the minimum concentration of *Salmonella*, which is 3.7 × 10^1^ cfu/mL.

### 3.4. Other Applications

In addition to biomedical, environmental, and food analyses, paper-based sensors with instrument-free signal readout have found utility in various other fields. For instance, Dias et al. [6] designed a paper-based sensor that was connected to a distance signal readout to observe acid-base titrations. The titrant and indicator were added to the paper and produced a visible paper channel. Then, the solution was delivered into the detection zone, where different concentrations of titrant would lead to different distances in the detection zone, thereby causing a change in the color or discoloration of the channel. Finally, this proposed paper-based sensor was utilized to evaluate the acetic acid levels of commercially available vinegars ranging from 4.3 to 4.6%. Inorganic carbon that is dissolved in water has a major influence on the balance of the carbon cycle on Earth. A paper apparatus that relies on distance signal readout was designed by Giménez-Gómez et al. [71] for the detection of dissolved inorganic carbon in freshwater. Initially, the titration reagents are introduced to the detection area, after which the sample is propelled up the whole area via capillary action, resulting in a color change of the indicator from yellow to blue. Finally, the length of the colored band can be recorded by a ruler. The developed device is able to detect DIC levels from 50 mg/L to 1000 mg/L.

## 4. Conclusions

In this review, we summarized some kinds of paper-based sensors with an instrument-free signal readout, including distance-based signal readout technology, counting-based signal readout technology, text-based signal readout technology, and other transduction technologies. On the other hand, the applications were also described based on these instrument-free signal readout technologies, such as biomedical analysis, environmental pollutant monitoring, and food safety analysis. However, each instrument-free signal readout technology has its own advantages and disadvantages. For example, distance-based signal readout technology has the advantage of high sensitivity, but it also has long analysis times and a narrow linear range. Count-based signal readout technology has the advantage of short analysis times, but it has low detection sensitivity and a narrow detection range. Time-based signal readout technology has the advantage of a wide detection range, but it suffers from long analysis times and low detection sensitivity. Thus, paper-based sensors with instrument-free signal readout technology still face some challenges and limitations: (1) To fulfill the requirements of more intricate, low-level, or multi-component samples, it is imperative to boost the accuracy and precision of paper-based sensors. (2) To make large-scale applications, paper-based sensors must be standardized and commercialized. Thus, it is necessary to establish unified testing standards and quality control procedures, and develop commercial products suitable for different fields. (3) The capability of paper-based sensors to perform multiple channels of operation makes them suitable for more complex analysis and detection tasks.

Additional research and development are required for paper-based sensors with an instrument-free signal readout in the future aiming to achieve the following: (1) explore new paper materials and preparation technologies to achieve higher sensitivity, selectivity, and stability; (2) paper-based sensors can be integrated with other sensor technologies, including optical, electrochemical, and multimodal analysis, to create a comprehensive sensing system; (3) automation and digitization technologies can be utilized to enhance operational convenience, repeatability, and data-processing capabilities of paper-based sensors. In summary, paper-based sensors with instrument-free signal readout have great potential in scientific research and practical applications. It is anticipated that paper-based sensors will become more accessible in the future as technological challenges are addressed and further research is conducted.

## Figures and Tables

**Table 1:** Application of paper-based sensors with instrument-free signal readout technologies in biomedical analysis.

Analytes/Real Sample	Composition/Design Method	Signal Readout Technologies	Limit of Detection	Linear Range	Ref.
Adenosine ; Interferon-γ; Pb^2+^/Human serum; Water sample	A circular region a and b, a rectangular c	Distance-based technology	Adenosine: 1.6 µM; Interferon-γ: 0.3 nM; Pb^2+^: 0.5 nM	Adenosine: 1.7–62.5 µM; Interferon-γ: 0.5–32 nM; Pb^2+^: 0.75–50 nM	[39]
SARS-CoV-2/Nasal swab	/	Distance-based technology	1 ng/mL	1–10 μg/mL	[40]
Glucose/Tears	A sample zone, primary circular zone, detection zone, and circular absorption zone/A wax printed method	Distance-based technology	0.1 mM	0.1–1.2 mM	[41]
Hemoglobin A1c/Human blood	The C line and T line, absorbent pad, and sample pad	Distance-based technology	-	3.3–15.1%	[42]
Interleukin-6 (IL-6)/Human saliva and urine	Circular sample zone, capture zone, and detection channel zone/A wax printed method	Distance-based technology	0.05 pg/mL	0.05–25.0 pg/mL	[43]
Urinary albumin (Alb) and creatinine (Cre)/Human urine	Sample inlet area and detection area/A wax printed method	Distance-based technology and Text-based technology	-	Alb: 0–1000 mg/mL; Cre: 0–3000 mg/mL	[44]
Chymotrypsinogen/Human urine	A sample application zone, a detection zone, and an absorbent zone/A wax printed method	Distance-based technology	3.5 μM	2.4–29.2 μM	[45]
Glucose and uric acid/Serum and urine	Detection layer and the auxiliary layer/A wax printed method	Other transduction technology	Glucose: 3 μM; Uric acid: 4 μM	Glucose: 0.01–10 mM; Uric acid: 0.01–5 mM	[46]
Doxycycline hyclate (DOX) and oxymetazoline hydrochloride (OXY)/Doxycycline^®^ tablets and Oxymetazoline^®^ Nasal drops	Reaction zones/A wax printed method	Other transduction technology	-	DOX: 0.5–5 mg/L; OXY: 1.0–40 mg/L	[47]
Albumin (Alb) and Alkaline phosphatase (ALP)/Human serum	Sample inlet zone, pretreatment zone, detection zone/A wax printed method	Distance-based technology	Alb: 0.8 g/L; ALP: 5 U/L	Alb: 1–25 g/L; ALP: 5–50 U/L; 50–200 U/L	[48]
Ascorbic acid and captopril/Vitamin C and captopril tablets	The circular zone and a straight channel/A wax printed method	Distance-based technology	Ascorbic acid: 8.4 × 10^−4^ M; Captopril: 1.1 × 10^−3^ M	Ascorbic acid: 0.001–0.012 M; Captopril: 0.001–0.01 M	[49]
Oxalate/Urine sample	Sample loading zone, test zone and absorbent paper pad	Counting-based technology	0.3 mM	0.3–0.7 mM	[50]

**Table 2:** Application of paper-based sensors with instrument-free signal readout technologies in environmental analysis and food safety.

Analytes/Real Sample	Composition/Design Method	Signal Readout Technology	Limit of Detection	Linear Range	Ref.
Chloride/Tap water	A straight channel and a circular zone/A wax printed method	Distance-based technology	Cl^−^: 1.7 mg/L	Cl^−^: 5–200 mg/L	[56]
Tetracycline/Water and soil extracts	/	Other transduction technology	Tetracycline in water: 5.23–17.1 μg/L; Tetracycline in soil extracts: 5.21–35.3 μg/kg	Tetracycline in water: 75–10,000 μg/L; Tetracycline in soil extracts: 75–7500 μg/L	[57]
Cu^2+^, Pb^2+^, and Ag^+^/Tap water and Fresh water	Chamber 1, chamber 2, and trapping channel	Distance-based technology	Cu^2+^: 103.1 nM; Pb^2+^: 69.5 nM; Ag^+^: 793.6 nM	Cu^2+^: 0–100 nM; Pb^2+^: 0–100 nM; Ag^+^: 0–1000 nM	[58]
Ni^2+^/Tap water and Mineral water	A sample introduction zone and four circular detection zones/A wax printed method	Counting-based technology	-	0–8 mM	[59]
Al^3+^/Gold King Mine Water Samples	Sample inlet, the channel, and detection zone/A wax printed method	Distance-based technology	2.5 ppm; 0.9 ppm	2–54 ppm; 2–24 ppm	[60]
Polygalacturonase/Cucumber sample	/	Distance-based technology	0.025 U/mL	0.025–0.80 U/mL	[61]
Ascorbic acid/Orange juice and Vitamin C tablets	A rectangular reservoir, two straight parallel channels, a hydrophobic space, and a 3D connector/A wax printed method	Distance-based technology	16 µM	0.05–1.2 mM	[62]
Ascorbic acid/Beverages	Sample loading area, microfluidic channel, a detection zone/A screen printed technique	Other transduction technology	2.5 mg/L	2.5–1000 mg/L	[63]
Sulfonamides/Cow milk	Four microchannels containing two spots connected by the sample application chamber/A wax printed method	Other transduction technology	Sulfamethazine: 2.80 μM; Sulfadimethoxine: 2.70 μM; Sulfathiazole: 2.50 μM	2.5–40.0 μM	[64]
Zn^2+^/Drinking water, dietary supplements, micronutrient fertilizer	Loading zone and detection zone/A wax printed method	Distance-based technology	1.87 nM; 1.32 nM; 1.57 nM	0–30.0 nM; 0–60.0 nM; 0–70.0 nM	[65]

## Data Availability

Not applicable.
